# Heat Capacity and Absolute Standard Entropy of the High Temperature Polymorph of Calcium Boranate and Thermodynamic Calculations Regarding its Decomposition and Rehydrogenation

**DOI:** 10.1002/cphc.202500108

**Published:** 2025-11-12

**Authors:** Konrad Burkmann, Franziska Habermann, Alexander Walnsch, Bianca Störr, Jürgen Seidel, Klaus Bohmhammel, Roman Gumeniuk, Florian Mertens

**Affiliations:** ^1^ Institut für Physikalische Chemie TU Bergakademie Freiberg Lessingstraße 45 Freiberg 09599 Germany; ^2^ Institut für Werkstoffwissenschaft TU Bergakademie Freiberg Gustav‐Zeuner‐Straße 5 Freiberg 09599 Germany; ^3^ GTT‐Technologies Kaiserstraße 103 Herzogenrath 52134 Germany; ^4^ Institut für Experimentelle Physik TU Bergakademie Freiberg Leipziger Straße 23 Freiberg 09599 Germany; ^5^ Zentrum für effiziente Hochtemperatur‐Stoffwandlung TU Bergakademie Freiberg Winklerstraße 5 Freiberg 09599 Germany

**Keywords:** absolute entropy, calcium boranate, calorimetry, heat capacity function, hydrogen storage, thermodynamics

## Abstract

Calcium boranate (Ca(BH_4_)_2_) is synthesized using wet chemistry metathesis reactions resulting in mixtures of both *α*‐ and β‐Ca(BH_4_)_2_, with the β phase being the main component. The drying procedure reveals high kinetic stability of the high temperature β polymorph, which is in contrast to the expectations based on the literature. The molar heat capacity function of β‐Ca(BH_4_)_2_ is determined between 2 and 525 K using different calorimeters, a Physical Property Measurement System applying the relaxation method in the low temperature range and a Calvet‐DSC for the high temperature range. From these values the absolute standard entropy at 298.15 K for β‐Ca(BH_4_)_2_ is calculated as *S* ^°^(298.15 K) = (117.4 ± 4.1) J mol^−1^ K^−1^. Taking the value of the enthalpy of formation from the literature, the Gibbs energy functions are calculated and the decomposition and rehydrogenation behavior of the compound is discussed.

## Graphical abstract

The decomposition route and rehydrogenation ability as well as the behavior in the low temperature range of β‐Ca(BH_4_)_2_ synthesized by a metathesis reaction are investigated based on heat capacity measurements over a broad temperature range using two different calorimetric techniques.

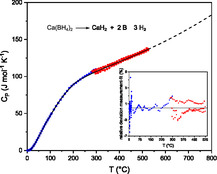

## 1 Introduction

Metal hydrides and their derivatives are still in the focus of research as candidates for hydrogen storage materials.^[^ ^1^, ^2^, ^3^, ^4^, ^5^, ^6^, ^7^, ^8^, ^9^, ^10^ ^]^ Besides high entropy alloys^[^ ^5^, ^6^, ^7^ ^]^ and metal alanates,^[^ ^3^, ^6^, ^9^, ^11^, ^12^, ^13^ ^]^ metal boranates^[^ ^4^, ^6^, ^9^, ^10^, ^14^, ^15^, ^16^, ^17^ ^]^ are of high interest to the hydrogen storage community because of their potential use in the design of hydrogen storage systems based on thermodynamic tuning.^[^ ^18^, ^19^ ^]^ To find, evaluate, and optimize such systems, their decomposition behavior with and without reactive additives (mostly metal hydrides like MgH_2_^[^ ^19^, ^20^, ^21^, ^22^, ^23^, ^24^ ^]^ but also other complex hydrides^[^ ^23^, ^24^, ^25^, ^26^, ^27^, ^28^, ^29^, ^30^, ^31^, ^32^, ^33^, ^34^ ^]^) can be calculated from their basic thermodynamic properties, such as their heat capacity function, absolute standard entropy, enthalpy of formation, phase transition data and others, as recently demonstrated by Palumbo et al.^[^ ^8^ ^]^ for different hydrides.

Ca(BH_4_)_2_ is an attractive candidate not only in terms of hydrogen storage,^[^ ^10^ ^]^ but also as a material for electrochemical applications in batteries,^[^ ^10^, ^35^, ^36^, ^37^, ^38^ ^]^ as a reducing agent and catalyst in organometallic and organic synthesis^[^ ^10^, ^39^ ^]^ and even for the design of high temperature superconductors.^[^ ^40^ ^]^ There are a lot of experimental investigations focusing on the phase transitions, including related structural details (e. g.^[^ ^31^, ^41^, ^42^, ^43^, ^44^, ^45^ ^]^) and on the decomposition and rehydrogenation behavior (e. g.^[^ ^10^, ^42^, ^43^, ^46^, ^47^, ^48^, ^49^, ^50^, ^51^, ^52^, ^53^, ^54^ ^]^) including catalyzed systems.^[^ ^46^, ^53^ ^]^ Unfortunately, there are only three experimental investigations^[^ ^22^, ^51^, ^55^ ^]^ focusing on the thermodynamic quantities resulting in an insufficient data basis for Ca(BH_4_)_2_, hence accurate experimental determination of these quantities are required to understand the observed behavior of Ca(BH_4_)_2_ in the investigated mixtures^[^ ^21^, ^22^, ^23^, ^24^, ^25^, ^26^, ^27^, ^28^, ^29^, ^30^, ^31^ ^]^ and to design of such reactive hydride mixtures.

Ca(BH_4_)_2_ can be synthesized using several well‐established routes:^[^ ^4^, ^56^ ^]^ a) reaction of calcium tetramethoxy borate with diborane,^[^ ^52^ ^]^ b) reaction of calcium methylate with diborane,^[^ ^57^ ^]^ c) reaction of CaH_2_ with BH_3_‐solvent adducts,^[^ ^55^, ^58^, ^59^, ^60^ ^]^ d) reaction of calcium dihydride with calcium hexaboride and pressurized hydrogen,^[^ ^53^, ^61^ ^]^ and e) reaction of calcium chloride with various alkali metal boranates and various solvents^[^ ^31^, ^39^, ^62^, ^63^, ^64^ ^]^ analogously to reaction Equation (1). Of course, there are also unusual methods (e. g.^[^ ^22^, ^54^, ^65^, ^66^ ^]^), which are not explained in detail here.

(1) $${\text{CaCl}}_{\text{2}}+{\text{2 NaBH}}_{\text{4}}\;\overset{\text{[THF]}}{\to }\;\text{Ca}{\left({\text{BH}}_{\text{4}}\right)}_{\text{2}}+\text{2 NaCl}$$

The first three synthesis routes mentioned are based on the procedures described by Wiberg et al.^[^ ^52^, ^57^, ^58^ ^]^ in the 1960s. Method e) seems to be the most efficient one because of the little effort necessary to remove the by‐product and the low cost for the raw materials compared to CaH_2_. The ease of the by‐product removal is due to the poor solubility of NaCl in tetrahydrofurane (THF).^[^ ^67^ ^]^ An additional benefit comes from the simplicity of the handling of dissolved compounds compared to that of gaseous species, in this case diborane. The removal of THF from the solvent adduct complex results in β‐Ca(BH_4_)_2_ which was then used for calorimetric measurements to determine some of its thermodynamic quantities.

Within this study, we present the heat capacity function of β‐Ca(BH_4_)_2_ over a wide temperature range starting from about 2 K. From these data, the Debye temperature and the absolute standard entropy of the compound were calculated. In combination with literature values, they were also used to calculate possible decomposition routes and to draw conclusions about the decomposition and rehydrogenation capability.

## 2 Results and Discussion

### 2.1 Synthesis and Characterization of β‐Ca(BH_4_)_2_

**Figure 1:**
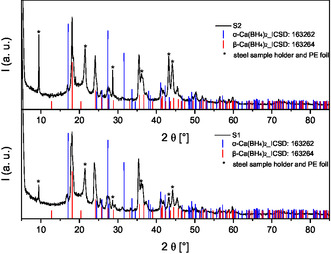
Powder X‐ray diffraction pattern of the synthesized products containing α‐ and β‐Ca(BH_4_)._2_ (top: S2; bottom: S1). The included reference reflections are taken from the ICSD.^[^ ^68^ ^]^

**Figure** **1** shows the X‐ray powder diffractograms of the residues obtained after removal of the solvent of the centrifuged reaction solution and applying the described drying procedures. The comparison with reflections of the reference material taken from the literature^[^ ^68^ ^]^ indicates that the two samples consist both of α‐ and β‐Ca(BH_4_)_2_. Although using several other drying procedures described in refs., [31, 41, 63, 64, 69, 70] it was not possible to synthesize either the pure room temperature α phase or the pure high temperature β phase. It seems that the β phase was the main component in all experiments performed. To quantify the content of both phases, a Rietveld analysis was carried out using the sample S1, delivering a value of about 3% for the content of the α phase, which is below the typical uncertainty for this type of analysis of about 5%. Its content in the sample S2 was expected to be even smaller, because the drying procedure used leads to the formation of the β phase. Despite the presence of the α phase, we nevertheless used these samples for calorimetric measurements, because the amount is rather small and the difference between the heat capacity of both phases can be assumed to be small, too.

**Figure 2:**
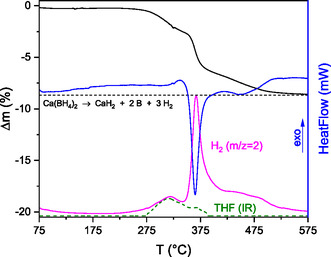
TG‐DSC‐FTIR/MS measurement of the sample S2. The horizontal dashed line represents the theoretical mass loss of the decomposition reaction Equation (2) according to ref. [49, 50, 52].

The thermogravimetry differential scanning calorimetry coupled with Fourier transform infrared as well as mass specttroscopy (TG‐DSC‐FTIR/MS) curves are presented in **Figure** **2**. It can be seen that sample S2 contains only traces of THF after desolvation, which do not reduce the sample purity significantly. Furthermore, it is possible to assign the experimental mass loss to the decomposition reaction Equation (2) (theoretical weight loss approx. −8.7%; see horizontal line in Figure 2).

(2) $$\text{Ca}{\left({\text{BH}}_{4}\right)}_{2}\to {\text{CaH}}_{2}+\text{2 B}+{\text{3 H}}_{2}$$

Wiberg et al.^[^ ^52^ ^]^ and Yan et al.^[^ ^49^, ^50^ ^]^ also suggest a decomposition of Ca(BH_4_)_2_ into CaH_2_, 2B, and 3H_2_ under these conditions.

### 2.2 Determination of the Heat Capacity Function of β‐Ca(BH_4_)_2_

As already mentioned, heat capacities were determined using different types of calorimeters: A Physical Property Measurement System (PPMS) was used in the range from 2 K to 298 K (values see **Table** **1**) and a Calvet type DSC in the range from 283 K to 525 K. The values corresponding to the first DSC cycle are given in **Table** **2** and those of the following three cycles in **Table** **3**.

**Table 1:** Heat capacity values of β‐Ca(BH_4_)_2_ (sample S1, M = 69.76 gmol^−1^) between 2 and 298 K determined using a PPMS DynaCool‐12 device. The uncertainties of the temperatures are *u*(T) = 0.01 K for 1.8 < T/K < 20, *u*(T) = 0.02 K for 20 < T/K < 100, and *u*(T) = 0.05 K for 100 < T/K < 300. The estimated relative combined expanded uncertainties are U(C_P_) = 0.08 for 2 < T/K < 10 and U(*C*_P_) = 0.02 for 10 < T/K < 298.

*T* [K]	*C*_P_ [Jmol^−1^ K^−1^]	*T* [K]	*C*_P_ [Jmol^−1^ K^−1^]	*T* [K]	*C*_P_ [Jmol^−1^ K^−1^]	*T* (K]	*C*_P_ [Jmol^−1^ K^−1^]
1.82	0.004367	6.62	0.1325	11.43	0.6443	16.17	1.700
1.92	0.004968	6.72	0.1381	11.53	0.6596	16.27	1.733
2.02	0.005555	6.82	0.1437	11.63	0.6767	16.38	1.759
2.12	0.006242	6.92	0.1490	11.73	0.6938	16.48	1.790
2.22	0.006806	7.02	0.1562	11.83	0.7107	16.58	1.821
2.32	0.007762	7.12	0.1635	11.93	0.7284	16.68	1.850
2.42	0.008529	7.22	0.1702	12.03	0.7456	16.79	1.880
2.52	0.009561	7.32	0.1774	12.13	0.7637	16.89	1.913
2.62	0.01053	7.42	0.1833	12.23	0.7822	17.24	2.035
2.72	0.01162	7.52	0.1895	12.33	0.8018	22.26	3.825
2.82	0.01273	7.62	0.1974	12.44	0.8201	27.38	6.056
2.92	0.01391	7.72	0.2052	12.54	0.8399	32.42	8.529
3.02	0.01520	7.82	0.2130	12.64	0.8600	37.46	11.22
3.12	0.01630	7.92	0.2209	12.74	0.8790	42.50	14.08
3.22	0.01782	8.02	0.2290	12.84	0.8994	47.54	17.05
3.32	0.01951	8.12	0.2367	12.94	0.9183	52.58	20.10
3.42	0.02113	8.22	0.2461	13.04	0.9399	57.63	23.17
3.52	0.02291	8.32	0.2548	13.14	0.9595	62.67	26.30
3.62	0.02484	8.42	0.2644	13.24	0.9813	67.71	29.42
3.72	0.02623	8.52	0.2743	13.34	1.003	72.74	32.34
3.82	0.02876	8.61	0.2825	13.44	1.023	77.78	35.23
3.92	0.03057	8.72	0.2914	13.54	1.045	82.83	38.04
4.02	0.03305	8.82	0.3021	13.64	1.068	87.88	40.67
4.12	0.03512	8.91	0.3130	13.74	1.090	92.93	43.28
4.22	0.03685	9.02	0.3209	13.84	1.111	97.97	45.94
4.32	0.04002	9.12	0.3309	13.94	1.134	103.0	48.69
4.42	0.04232	9.22	0.3428	14.04	1.158	106.1	50.25
4.52	0.04511	9.32	0.3546	14.14	1.181	115.7	55.24
4.62	0.04797	9.42	0.3652	14.24	1.206	125.3	59.95
4.72	0.05140	9.52	0.3791	14.35	1.227	134.9	64.20
4.82	0.05425	9.62	0.3888	14.45	1.253	144.5	68.51
4.92	0.05760	9.72	0.3989	14.55	1.277	154.0	72.70
5.02	0.05983	9.82	0.4125	14.65	1.301	163.6	76.63
5.12	0.06367	9.91	0.4620	14.75	1.330	173.2	80.40
5.22	0.06675	10.03	0.4387	14.85	1.350	182.8	84.24
5.32	0.07081	10.13	0.4518	14.95	1.377	192.4	86.97
5.42	0.07515	10.23	0.4654	15.05	1.403	201.9	89.33
5.52	0.07876	10.33	0.4788	15.16	1.429	211.5	91.36
5.62	0.08358	10.43	0.4928	15.26	1.455	221.1	93.98
5.72	0.08730	10.53	0.5063	15.36	1.481	230.7	96.06
5.82	0.09110	10.63	0.5202	15.46	1.507	240.3	98.13
5.92	0.09645	10.73	0.5357	15.56	1.535	249.8	100.0
6.02	0.1010	10.83	0.5512	15.66	1.562	259.4	101.8
6.12	0.1054	10.93	0.5646	15.76	1.588	269.0	103.5
6.22	0.1103	11.03	0.5795	15.87	1.618	278.6	105.8
6.32	0.1159	11.13	0.5958	15.97	1.643	288.1	108.6
6.42	0.1215	11.23	0.6115	16.07	1.674	297.9	109.7
6.52	0.1252	11.33	0.6265				

**Table 2:** Heat capacity values of β‐Ca(BH_4_)_2_ (sample S2, *M* = 69.76 gmol^−1^) between 283 K and 519 K determined in the first measurement cycle using a DSC 111. The uncertainty of the temperature is *u*(T) = 0.1 K for 283 < T/K < 519. estimated relative combined expanded uncertainty is U(*C*_P_) = 0.03 for 283 < T/K < 519.

*T* [K]	*C*_P_ [Jmol^−1^ K^−1^]	*T*[K]	*C*_P_ [Jmol^−1^ K^−1^]	*T* [K]	*C*_P_ [Jmol^−1^ K^−1^]	*T* [K]	*C*_P_ [Jmol^−1^ K^−1^]
282.7	111.3	340.3	119.3	409.6	135.1	469.0	135.1
288.8	112.6	350.1	121.5	419.7	155.5	478.9	135.7
294.8	113.6	360.1	123.1	429.6	155.6	488.8	136.8
300.8	114.0	370.0	124.3	439.3	138.7	498.7	137.9
310.4	114.9	379.9	126.4	449.2	133.3	508.6	139.0
320.3	117.2	389.7	129.9	459.1	133.9	518.5	140.6
330.3	119.1	399.7	132.1				

**Table 3:** Heat capacity values of β‐Ca(BH_4_)_2_ (sample S2, *M* = 69.76 gmol^−1^) between 283 K and 525 K determined in the second, third and fourth measurement cycle using a DSC 111. The uncertainty of the temperature is *u*(T) = 0.1 K for 283 < T/K < 525. The estimated relative combined expanded uncertainty is U(*C*_P_) = 0.03 for 283 < T/K < 519.

*T* [K]	*C*_P_ [Jmol^−1^ K^−1^]	*T* [K]	*C*_P_ [Jmol^−1^ K^−1^]	*T* [K]	*C*_P_ [Jmol^−1^ K^−1^]	*T* [K]	*C*_P_ [Jmol^−1^ K^−1^]
282.6	106.9	346.5	110.1	409.6	123.5	469.1	130.3
288.8	108.0	350.1	115.3	415.5	121.2	475.0	129.1
294.8	108.9	356.1	111.8	415.7	121.6	475.1	128.9
296.3	106.2	356.4	111.5	419.5	124.6	479.0	131.6
296.5	104.1	360.1	116.5	425.4	122.5	484.8	130.5
300.8	109.3	366.1	114.0	425.6	122.6	484.9	130.0
306.4	107.0	366.3	114.0	429.5	125.8	488.8	133.0
306.6	104.8	370.0	117.7	435.4	123.4	494.7	132.1
310.4	110.5	375.9	115.1	435.5	123.7	494.8	131.3
316.5	108.2	376.1	115.3	439.3	126.9	498.8	134.4
316.8	106.5	379.9	118.6	445.3	124.8	504.7	133.6
320.3	111.4	385.8	116.8	445.3	125.2	504.7	132.3
326.3	109.6	386.0	116.6	449.2	127.8	508.7	135.9
326.5	108.5	389.8	120.2	455.2	126.1	514.4	135.0
330.2	112.8	395.7	118.1	455.3	126.7	514.6	133.8
336.3	108.2	395.9	118.3	459.1	129.1	518.6	138.1
336.5	108.6	399.7	121.9	465.0	127.6	524.4	137.0
340.3	113.5	405.7	119.5	465.2	127.8	524.5	135.5
346.2	110.1	405.8	119.8				

**Figure 3:**
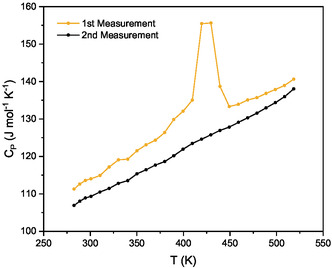
First and second heat capacity measurement run of sample S2 showing the polymorphic transition of the α to the β phase of Ca(BH_4_)_2_.

A phase transition between the α and the β phase is visible in the first cycle of the DSC measurement (see **Figure** **3**). Unfortunately, there is no literature value for the enthalpy or entropy of transition and therefore, the content of the α phase cannot be calculated for a comparison with the Rietveld results. However, a phase transition temperature can be determined delivering a value of *T*_onset_ = 106 °C and *T*_peak_ = 147 °C, which is significantly lower than the literature values of about 106 °C.^[^ ^60^ ^]^ These lower values explain the formation of the β phase during the drying procedure applied to both samples, S1 and S2, which took place at 106 °C and 180 °C, respectively.

**Figure 4:**
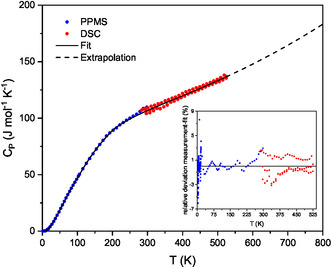
Molar heat capacity of β‐Ca(BH_4_)_2_ within the temperature range of 2 K to 525 K determined by relaxation method (blue dots, values see Table 1) and three independent DSC measurements (red dots, values see Table 3) as well as the fitted curve (black line). The relative deviation between fit and measurement is given in the insert.

In the following DSC measurement runs, the peak in the *C*_P_ curve was not present, indicating that the formation of the β phase is an irreversible process. Therefore, the second, third, and fourth DSC measurements were used for the fit of the heat capacity (see **Figure** **4**). Due to the good agreement between the values of the measurements carried out using the different calorimetric techniques in the overlapping region, the obtained values can be regarded as reliable.

For the fitting procedure, the entire temperature range has been divided into five parts where the data are fitted using the well‐established Maier–Kelley‐type polynomials^[^ ^71^ ^]^ (see Equation (3)).

(3) $$\frac{C_{P}}{\left[J\;{\text{mol}}^{-1}\;K^{-1}\right]}=a+b\cdot \frac{T}{\left[K\right]}+c\cdot \frac{T^{-2}}{\left[K^{-2}\right]}+d\cdot \frac{T^{2}}{\left[K^{2}\right]}+e\cdot \frac{T^{3}}{\left[K^{3}\right]}$$

The values of the fit coefficients, the coefficients of determination *R* ^2^, and the fit standard error *FitStdErr*, are presented in **Table** **4**. Depending on the significance of the different terms in the different temperature ranges, some coefficients were set to be zero to include only the relevant terms.

**Table 4:** Heat capacity values of β‐Ca(BH_4_)_2_ (sample S2, *M* = 69.76 gmol^−1^) between 283 K and 525 K determined in the second, third and fourth measurement cycle using a DSC 111. The estimated relative combined expanded uncertainty is U(*C*_P_) = 0.03 for283 < T/K < 525.

*T* range [K]	0–4	4–15	15–110	110–220	220–525
*a*	0	2.762E‐1	−6.929E0	−2.153E1	9.976E1
*b*	9.073E‐4	‐8.096E‐2	3.524E‐1	8.173E‐1	1.723E‐2
*c*	0	‐1.056E0	4.928E2	0	−7.288E5
*d*	0	8.752E‐3	4.268E‐3	−1.329E‐3	1.107E‐4
*e*	4.511E‐4	1.068E‐4	−2.390E‐5	0	0
*R* ^2^	0.9998	1.000	1.000	0.9997	0.9901
*FitStdErr* (J mol^−1^ K^−1^)	1.243E‐4	3.759E‐3	1.059E‐1	3.274E‐1	1.695E0

### 2.3 Absolute Standard Entropy and Entropy of Formation at 298.15 K of β‐Ca(BH_4_)_2_

The calculation of the absolute standard entropy at 298.15 K was performed by an integration of the *C*_P_(T)/*T* curve between 0 K and 298.15 K according to Equation (4), resulting in a value of *S* ^°^(298.15 K) = (117.4 ± 4.1) Jmol^−1^ K^−1^.

(4) $$S^{{}^{\circ}}(298.15\;K)=\int_{0\;K}^{298.15\;K}\frac{C_{P}}{T}dT$$

A calculation based on the integration of the estimated *C*_P_/*T* function, whereby the *C*_P_ function originates from a modified Neumann Kopp rule was also discussed in the literature.^[^ ^72^ ^]^ Pinatel et al.^[^ ^73^ ^]^ as well as Dematteis et al.^[^ ^55^ ^]^ calculated the heat capacity of “BH_4_” from Na and NaBH_4_ and added these values to the heat capacities of Mg and Ca. However, this method seems to be pointless, since the *C*_P_ values derived by this procedure for Mg(BH_4_)_2_ and Ca(BH_4_)_2_ deviate significantly from the experimental ones.^[^ ^55^ ^]^ In our previous study concerning Y(BH_4_)_3_, we found a similar behavior resulting in a large deviation of about 9% of the estimated absolute entropy value at 300 K from the one based on the calorimetric experiments.^[^ ^14^ ^]^

Using the absolute standard entropy values of Ca, B, and H_2_ to be found in the HSC database,^[^ ^74^ ^]^ the standard entropy of formation of β‐Ca(BH_4_)_2_ at 298.15 K has been calculated using Equation (5), resulting in a value of Δ_F_ *S* ^°^(298.15 K) = −458.7 Jmol^−1^ K^−1^.

(5) $$\Delta_{F}S_{\text{Ca}{\left({\text{BH}}_{4}\right)}_{2}}^{{}^{\circ}}(298.15\;K)=S_{\text{Ca}{\left({\text{BH}}_{4}\right)}_{2}}^{{}^{\circ}}(298.15\;K)-S_{\text{Ca}}^{{}^{\circ}}(298.15\;K)-2\cdot S_{B}^{{}^{\circ}}(298.15\;K)-4\cdot S_{H_{2}}^{{}^{\circ}}(298.15\;K)$$

### 2.4 Low Temperature Heat Capacity of β‐Ca(BH_4_)_2_

The general physical interpretation of heat capacities in the low temperature region usually takes into account, besides the Debye model representing the phonon contributions, electronic contributions as well (see Equation (6)).^[^ ^75^, ^76^, ^77^ ^]^

(6) $$\frac{C_{P}}{\left[{\text{Jmol}}^{-1}K^{-1}\right]}=\gamma \cdot \frac{T}{\left[K\right]}+\beta \cdot \frac{T^{3}}{\left[K^{3}\right]}$$

Usually, a significant electronic contribution is only expected from electrically conductive materials;^[^ ^75^, ^76^, ^77^ ^]^ however, the β‐Ca(BH_4_)_2_ is nonconductive. It is already known from other nonconductive materials, such as lithium iron phosphate,^[^ ^78^ ^]^ alkaline earth alanates,^[^ ^11^, ^12^, ^13^ ^]^ and boranates such as Sr(BH_4_)_2_^[^ ^15^ ^]^ and Y(BH_4_)_3_^[^ ^14^ ^]^ that an electronic contribution can appear because of defects in the crystal lattice, such as vacancies, impurities, and dislocations.^[^ ^78^, ^79^ ^]^ This situation may also be applicable to this compound due to the process of desolvation that can lead to such structural defects.

Fitting the heat capacity in the temperature range from 0 K and 4 K using [Equation (6)], the coefficients *γ* and β were determined as 9.073 × 10^−4^ Jmol^−1^ K^−1^ and 4.511 × 10^−4^ Jmol^−1^ K^−4^, respectively. Accepting that assumption, the Debye temperature, given by^[^ ^75^, ^76^, ^77^ ^]^

(7) $$\Theta_{D}=\sqrt[{3}]{\frac{12\cdot \Pi^{4}\cdot n\cdot R}{5\cdot \beta }}$$

possesses the value Θ_D_ = (361.9 ± 0.7) K. This is significantly higher than the corresponding one of Sr(BH_4_)_2_ ((273.9 ± 0.4) K)).^[^ ^15^ ^]^ This trend also exists for alkali boranates and apparently also for inorganic compounds containing tetrahedral anions and metal cations.^[^ ^80^ ^]^ Additionally, in the respective group of metals in the periodic table, the Debye temperature Θ_D_ or the frequency *v*_D_ of these compounds decreases almost without exception with increasing atomic number.^[^ ^80^ ^]^ An explanation can be derived from the characteristic phonon spectrum of boranates^[^ ^17^, ^81^, ^82^, ^83^, ^84^ ^]^ and alanates.^[^ ^85^ ^]^ The low‐energy region (branch) of the spectrum only reflects the displacement (vibrations) between the metal cation M^+^ and the complex anion ${\text{BH}}_{4}^{-}$.^[^ ^17^, ^81^, ^82^, ^83^, ^85^ ^]^ According to the theory of the heat capacity of solids, only these low‐energy phonons contribute to the heat capacity at low temperatures.^[^ ^75^, ^76^, ^77^ ^]^ The evaluation carried out in relation to the Debye temperature is limited to a temperature range from about 2 K to about 5 K. The Debye temperatures determined in this way therefore only reflect the interaction between the metal cation and the complex anion. The decrease in the Debye temperature from β‐Ca(BH_4_)_2_ to Sr(BH_4_)_2_ results from the inverse relationship between Θ_D_ and the molar mass, which was also indicated by Gavrichev et al.^[^ ^80^ ^]^

Furthermore, there seems to be a direct dependence between the Debye temperature and the Pauling electronegativity of the metal (Ca: 1.04 and Sr: 0.95^[^ ^86^ ^]^). According to the latter, the ionic bond is more pronounced in Sr(BH_4_)_2_ than in β‐Ca(BH_4_)_2_, which is reflected in the higher decomposition temperature for Sr(BH_4_)_2_ than for β‐Ca(BH_4_)_2_.^[^ ^4^, ^15^, ^56^ ^]^ The reciprocal correlation between the decomposition temperature and electronegativity of Pauling has already been demonstrated by several authors (i. e.^[^ ^6^, ^9^, ^83^, ^84^, ^87^, ^88^ ^]^). Having conducted a density functionial theory (DFT) study, Du et al.^[^ ^89^ ^]^ explain this phenomenon by the charge transfer from the complex anion to the cation, which is related to the electronegativity of the metal. Based on the present work, this statement can be extended to the reciprocal correlation between decomposition temperature and Debye temperature, at least in the case of alkaline earth boronates.

### 2.5 Enthalpy of Formation of β‐Ca(BH_4_)_2_

The determination of the enthalpy of decomposition from the DSC measurement in Figure 1 was not reliable due to the broad DSC signal and the large error associated with its integration. Therefore, the value of the enthalpy of formation cannot be calculated according to the procedure described in refs. [11, 12, 13, 16] However, there exists a DFT value for the enthalpy of formation of β‐Ca(BH_4_)_2_ of −366.158 kJmol^−1^ from Lee et al.^[^ ^26^ ^]^ that has been used for the following calculations.

### 2.6 Thermodynamic Calculations on the Deomposition and Rehydrogenation Behavior of β‐Ca(BH_4_)_2_

In this part, thermodynamic equilibrium calculations are presented regarding the decomposition products of β‐Ca(BH_4_)_2_ and their rehydrogenation ability using the determined values reported above for β‐Ca(BH_4_)_2_ and literature ones for all other compounds involved (see **Table** **5**). The heat capacity function polynomials used are of Maier–Kelley‐type (Equation (8)).^[^ ^71^ ^]^

(8) $$\frac{C_{P}}{\left[{\text{Jmol}}^{-1}K^{-1}\right]}=a+b\cdot \frac{T}{\left[K\right]}\cdot 10^{-3}+c\cdot \frac{T^{-2}}{\left[K^{-2}\right]}\cdot 10^{5}+d\cdot \frac{T^{2}}{\left[K^{2}\right]}\cdot 10^{-6}$$

**Table 5:** Thermodynamic data of compounds involved in the thermodynamic equilibrium calculations. Data taken from HSC 5.1,^[^ ^74^ ^]^ if not indicated further. The bold numbers mark the data determined within this study. The coefficients of the heat capacity function correspond to Equation (8).

compound	Δ_F_H^°^(298.15 K) [kJmol^−1^]	S^°^(298.15 K) [Jmol^−1^ K^−1^]	Coefficients of the Heat Capacity Function				
			T range [K]	*a*	*b*	*c*	d
$H_{2_{\text{(g)}}}$	0	130.679	298.15‐5000	25.855	4.837	1.584	−0.372
B	0	5.900	298.15‐1500	16.033	12.895	−7.570	−3.234
Ca	0	41.588	298.15‐716	16.311	22.218	2.673	−0.007
CaH_2_	−181.502	41.401	298.15‐1053	29.928	37.133	0	0
CaB_6_	−167.360^[^ ^114^ ^]^	56.230	300‐1200	86.980	136.994	−33.566	−57.877
β‐Ca(BH_4_)_2_	−366.158^[^ ^26^ ^]^	**117.4 ± 4.1**	**220‐524**	**99.760**	**17.233**	**−7.288**	**110.700**

At first, the thermodynamically favored decomposition reaction, including reaction equations Equation (9) to (11) taken from the literature,^[^ ^10^, ^25^, ^48^, ^49^, ^50^, ^51^, ^52^, ^90^, ^91^ ^]^ needs to be calculated.

(9) $$\text{Ca}{\left({\text{BH}}_{4}\right)}_{2}\to {\text{CaH}}_{2}+\text{2 B}+{\text{3 H}}_{2_{\text{(g)}}}$$

(10) $$\text{3 Ca}{\left({\text{BH}}_{4}\right)}_{2}\to {\text{2 CaH}}_{2}+\frac{1}{3}{\text{ CaB}}_{6}+\frac{10}{3}{\text{ H}}_{2(g)}$$

(11) $$\text{Ca}{\left({\text{BH}}_{4}\right)}_{2}\to {\text{CaB}}_{2}H_{6}+H_{2_{\text{(g)}}}$$

Unfortunately, there are no reliable thermodynamic data for CaB_2_H_6_ (see reaction equation Equation 11), so it could not be included in our calculations. Therefore, we focused on the first two reactions listed above and the reaction between CaH_2_ and boron to form CaB_6_ (see reaction equation Equation (12) as the second decomposition step after (Equation (9) as the first reaction).

(12) $$\frac{1}{3}{\text{ CaH}}_{2}+\text{2 B}\to \frac{1}{3}{\text{ CaB}}_{6}+\frac{1}{3}{\text{ H}}_{2_{\text{(g)}}}$$

**Figure 5:**
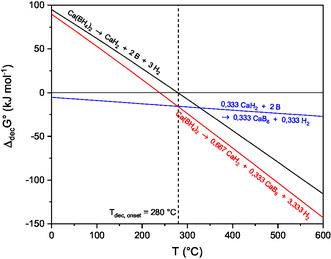
Thermodynamic calculations regarding the equilibrium decomposition behavior of β‐Ca(BH_4_)_2_ at 1 bar (black: reaction Equation (9); red: reaction Equation (10); blue reaction Equation (12)). The mentioned onset decomposition temperature was determined using the onset of the mass loss (see Figure 2).

**Figure 6:**
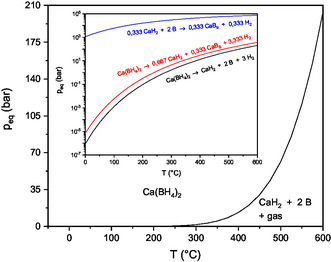
Thermodynamic calculations regarding the equilibrium hydrogen pressure for reaction Equation (9) calculated using Equation (13). The insert shows a logarithmic plot of the pressure against temperature for the reactions Equation (9), (10) and (12).

**Figure** **5** presents the calculated equilibrium decomposition behavior for reaction equations Equation (9), (10), and (12) depending on the temperature using a constant pressure of 1 bar. **Figure** **6** shows the temperature dependence of the equilibrium hydrogen pressure calculated, using Equation (13) for the experimentally observed decomposition reaction Equation (9).

(13) $$p=p^{{}^{\circ}}\cdot e^{-\frac{\Delta_{\text{dec}}G^{{}^{\circ}}}{R\cdot T\cdot \sum_{i}\nu_{\text{i,gas}}}}$$

Using the results of the calculations displayed in Figure 5, the observed experimental decomposition behavior is discussed in the following. The decomposition into CaH_2_, CaB_6_, and H_2_ is favored from a thermodynamically point of view, but in the experiment, no CaB_6_ has been identified using powder X‐ray diffractometry. In addition, the decomposition into CaH_2_, B, and H_2_ (see reation Equation (9)) is already possible at the measured decomposition onset temperature in agreement with our calculations. The decomposition may start with a melting of the compound, followed by a decomposition. Some authors observed this behavior, too.^[^ ^49^, ^50^, ^52^ ^]^ Furthermore, Yan et al.^[^ ^49^, ^50^ ^]^ found the presence of complex decomposition products during the decomposition, leading to the suggestion that after melting, the compound decomposes into an intermediate that later reacts to form CaH_2_, B, and H_2_.

The hydrogen pressure required for a rehydrogenation of the decomposition products as a function of the temperature is shown in Figure 6. From a thermodynamic point of view, it is possible to rehydrogenate the decomposition residue with technically feasible pressures, which is in agreement with other literature reports.^[^ ^49^ ^]^ It is likely that there are also kinetic obstacles that could not be taken into account in our calculations.

It is necessary to mention that the value for the enthalpy of formation of β‐Ca(BH_4_)_2_ used is a calculated one and therefore in addition to the uncertainty resulting from the experimental determined values is another source of uncertainty affecting the calculated Δ_dec_G(*T*) curves and thus the hydrogen pressures.

## 3 Conclusion

The high‐temperature phase of calcium boranate (β‐Ca(BH_4_)_2_) was synthesized using a wet chemical metathesis reaction. The determination of the decomposition route was carried out using simultaneous thermal analysis. In combination with powder X‐ray diffraction and Rietveld analysis, the phase composition and purity of the compound was demonstrated.

The heat capacity function covering a broad temperature range (2 K–525 K) was determined using calorimetric measurements. From these data, the absolute standard entropy was calculated to be *S* ^°^(298.15 K) = (117.4 ± 4.1) Jmol^−1^ K^−1^. Furthermore, the Debye temperature was determined from the coefficients of the heat capacity function at low temperatures, resulting in a value of (361.9 ± 0.7) K, which is higher than the respective value of Sr(BH_4_)_2_ with (273.9 ± 0.4) K.

Calculations regarding the thermodynamic decomposition behavior of β‐Ca(BH_4_)_2_ were performed using an enthalpy of formation value from the literature, which was calculated by DFT. The results of the calculations suggest a favored decomposition into CaB6. Surprisingly, this route has not been observed during the experiments, which is presumably caused by the presence of kinetic barriers. Furthermore, the experimentally observed decomposition route also appears as spontaneously possible from a thermodynamic point of view. There may be a melting of the boranate or a decomposition into intermediate compounds during the decomposition, before the final solid residue mixture is formed.

The calculations regarding the hydrogenation of the decomposed solid residue deliver comparable results with the literature. However, kinetic constraints were not considered within the present study, resulting in deviations between the experimental rehydrogenation behavior and the calculated one presented here.

Lacking an experimental value, a DFT calculated one was used for the enthalpy of formation of β‐Ca(BH_4_)_2_ in all thermodynamic calculations presented. Therefore, a certain degree of uncertainty within our calculations cannot be excluded.

A better understanding of the decomposition and rehydrogenation behavior of β‐Ca(BH_4_)_2_ was obtained using some literature data in addition to the own experimental ones. Future research should focus on the experimental determination of the enthalpy of formation of β‐Ca(BH_4_)_2_ and its possible use in reactive hydride mixtures with the goal of reducing the dehydrogenation temperature by simultaneously increasing the hydrogen pressure. The application of appropriate catalysts may be beneficial to overcome apparent kinetic obstacles.

## 4 Experimental Section

4.1

4.1.1

##### Materials

All handling and manipulation of the chemicals, as well as the sample preparation for the measurements, were carried out in an argon (Nippon Gases, 5 N) filled glove box with a gas circulation purifying system (H_2_O and O_2_ < 0.1 ppm) from MBraun or at an argon filled Schlenk line using Schlenk techniques.^[^ ^92^ ^]^ Sodium borohydride (NaBH_4_, 98%, ABCR) and calcium chloride (CaCl_2_, ACS; 96% min; anhydrous, Alfa Aesar) were dried as explained in ref. [12, 15] respectively. THF (C_4_H_8_O, VWR International GmbH, 99%) was dried over a 4 Å molecular sieve. A 25 μm thick Copper foil (Cu, 99.999%, Puratronic) and high‐purity granular Sapphire (aluminum oxide, Al_2_O_3_) were used within the heat capacity measurements without further purification.

##### Synthesis Procedures

Ca(BH_4_)_2_ was synthesized based on the procedure published by Chu et al.^[^ ^31^ ^]^ CaCl_2_ (7.5 mmol) and NaBH_4_ (14.9 mmol) were pre‐milled in a Fritsch Pulverisette 6 planetary ball mill device equipped with a 80 mL‐tungsten carbide (WC) crucible and three WC balls with an outer diameter of 10 mm and 7.1 g weight each ball (15 min milling and 5 min pause, total milling time of 1 h with a speed of 500 min^−1^). After that process, the powder was dissolved in 65 mL THF, forcing the formation of dissolved Ca(BH_4_)_2_ and suspended NaCl (see reaction Equation (1)).

The mixture was then stirred at room temperature for 5 d. The residue (NaCl) was removed by inert centrifugation because it was too fine for removal by inert filtration. This procedure has already been proven to be successful for Sr(BH_4_)_2_.^[^ ^15^ ^]^ The solvent of the centrifugate was distilled in vacuum into a cold trap, leaving a white residue consisting of Ca(BH_4_)_2_ × 1THF and Ca(BH_4_)_2_ × 2THF behind. A portion of this mixture was dried in a vacuum at 160 °C for 8 h followed by ice cooling (denoted as S1) and another portion was dried at 180 °C for 6 h without ice cooling (denoted as S2) according to the procedures described by Richter et al.^[^ ^60^ ^]^ In the first case, the α phase whereas in the second case, the β phase should be formed.

##### Powder X‐ray Diffraction (PXRD) and Rietveld Refinement

All diffractometric measurements were performed on a D2 PHASER diffractometer from Bruker equipped with a LYNXEYE detector in the range of 5° 2*θ* to 85° 2*θ* with a step size of 0.05° 2*θ* and a dwell time of 1 s. The power of the copper X‐ray tube is 300 W, delivering Cu‐*K*_α_ radiation (λ(*K*_α_) = 1.54184 Å). The powders were ground with an Agate mortar and pestle and placed on a steel metal sample holder and then covered with a PE foil to protect the sample against hydrolysis and oxidation during the measurements. This method has been demonstrated to be effective for other oxygen and moisture sensitive compounds, too (e.g.^[^ ^2^, ^11^, ^12^, ^13^, ^14^, ^15^, ^93^, ^94^, ^95^, ^96^, ^97^, ^98^, ^99^ ^]^). In order to determine the phases present in the investigated samples, whole pattern‐based refinements using the Rietveld method^[^ ^100^, ^101^ ^]^ were performed, applying the software TOPAS.^[^ ^102^ ^]^

##### Simultaneous Thermogravimetry‐ Differential Scanning Calorimetry Coupled with Fourier Transform Infrared and Mass Spectroscopy (TG‐DSC‐FTIR/MS)

The TG‐DSC‐FTIR/MS measurements with about 10 mg of sample were performed in corundum crucibles on a Sensys TGA‐DSC from SETARAM coupled with a Varian Fourier transform infrared spectrometer 3100 FT‐IR Excalibur and a Pfeiffer Omnistar quadrupole MS. A heating/cooling rate of 5 Kmin^−1^ and an argon purge gas flow of 20 Lmin^−1^ was used. The MS and FTIR served to determine the gaseous decomposition products, such as THF and hydrogen. The data processing was carried out with the Calisto v1.065, AKTS/SETARAM instrument software.

##### Calorimetric Heat Capacity Measurements

The heat capacities were measured in a temperature range from 2 to 525 K with different calorimeters depending on their specification.

The measurements in the low temperature region from 2 to 298 K were conducted with a PPMS (DynaCool‐12, Quantum Design) equipped with the heat capacity option according to the procedure described elsewhere.^[^ ^11^, ^12^, ^13^, ^14^, ^15^, ^78^, ^103^ ^]^ This method is based on a relaxation technique explained in ref. [104, 105] The preparation of the small copper crucibles (≈25 mg) containing the powdered sample (≈12 mg) used was done according to the procedure published by Loos et al.^[^ ^78^, ^106^ ^]^ as well as Shi et al.^[^ ^107^ ^]^ resulting in compressed samples of about 3 mm in height as well as in diameter. The copper crucibles were attached to the sample platform using Apiezon N grease to ensure good thermal contact between them. Furthermore, the Apiezon N grease possesses a low parasitic heat capacity.^[^ ^108^, ^109^ ^]^ The PPMS software automatically computes the heat capacity of the sample and crucible by subtracting the values of the addenda measurement (sample platform and grease) from the measured data of the sample (sample and crucible, sample platform, and grease).^[^ ^107^ ^]^ For the calculation of the molar heat capacity of the sample C_*P*,sample_ Equation (14) was used.

(14) $$C_{P\text{,sample}}=\frac{c_{\text{S,total}}-\omega_{\text{crucible}}\cdot c_{\text{S,crucible}}}{\omega_{\text{sample}}}\cdot M_{\text{sample}}$$

The difference between the total measured specific heat *c*_s,total_ and the specific heat of the copper crucible *c*_S,crucible_, which is calculated from mass‐weighted literature heat capacity values,^[^ ^110^ ^]^ delivers the specific heat of Ca(BH_4_)_2_. By multiplication by its molar mass *M*_sample_, the molar heat capacity of the sample C_*P*,sample_ was obtained.

The heat capacity measurements at temperatures higher than 280 K were performed in a DSC 111 from SETARAM using the instrument software Calisto v1.065, AKTS/SETARAM to conduct the data evaluation. The C_*P*_‐by‐step method^[^ ^111^ ^]^ used was demonstrated to be successfully applicable for many other compounds (see e. g. ref. [11, 12, 13, 14, 15, 16, 78, 93, 94, 96, 97, 98, 112]). Therefore, the same procedure was applied for the compound investigated within this study. In detail, about 85 mg of the sample was filled into a standard aluminum sample vessel. Different step programs using either 6 or 10 K steps with a heating rate of 3 Kmin^−1^ followed by an isothermal period of 60 min duration each were applied in the temperature range between 283 and 525 K. These temperature programs were used for the measurements of the sample, the reference (granular sapphire for the verification of the overall accuracy of the experiments), and the blank, which has been an empty aluminum vessel. Using the recommended reference heat capacity values of sapphire from Della Gatta et al.^[^ ^113^ ^]^ the heat capacities of the sample were calculated via Equation (15).

(15) $${\bar{C}}_{\text{P,sample}}=\frac{\int_{t_{i}}^{t_{i+1}}{\dot{Q}}_{\text{sample}}dt-\int_{t_{i}}^{t_{i+1}}{\dot{Q}}_{\text{blank}}dt}{\int_{t_{i}}^{t_{i+1}}{\dot{Q}}_{\text{ref}}dt-\int_{t_{i}}^{t_{i+1}}{\dot{Q}}_{\text{blank}}dt}\cdot \frac{m_{\text{ref}}}{m_{\text{sample}}}\cdot {\bar{c}}_{\text{S,ref}}\cdot M_{\text{sample}}$$

${\bar{C}}_{\text{P,sample}}$ and ${\bar{c}}_{\text{S,ref}}$ denote the molar and the specific heat of the sample and the reference material at the mean temperature of the ramp, respectively. The limits of the integration intervals *t*_i_ and *t*_i+1_ refer to the beginning and end of the peak of the heat flow *Q˙*. The symbols *m*_sample_ and *m*_ref_ describe the masses of the samples and the reference, respectively. *M*_sample_ denotes the molar mass of Ca(BH_4_)_2_.

## Conflict of Interest

The authors declare no conflict of interest.

## Data Availability

The data that support the findings of this study are available from the corresponding author upon reasonable request.

## References

[1] L. Sandig‐Predzymirska , J. Ortmeyer , J. Wagler , E. Brendler , F. Habermann , M. Anders , M. Felderhoff , F. Mertens , Dalton Trans. 2020, 49, 17689. 33232434 10.1039/d0dt03175e

[2] F. Habermann , K. Burkmann , B. Hansel , B. Störr , C. Schimpf , J. Seidel , M. Bertau , F. Mertens , Dalton Trans. 2023, 52, 4880. 36942882 10.1039/d2dt03718a

[3] K. Suárez‐Alcántara , J. R. Tena‐Garcia , R. Guerrero‐Ortiz , Materials 2019, 12, 2724. 31450714 10.3390/ma12172724 PMC6747775

[4] K. Suárez‐Alcántara , J. R. Tena García , Materials 2021, 14, 2561. 34069281 10.3390/ma14102561 PMC8156325

[5] F. Marques , M. Balcerzak , F. Winkelmann , G. Zepon , M. Felderhoff , Energy Environ. Sci. 2021, 14, 5191.

[6] Z. Cao , F. Habermann , K. Burkmann , M. Felderhoff , F. Mertens , Hydrogen 2024, 5, 241.

[7] M. Baricco , E. M. Dematteis , J. Barale , M. Costamagna , M. F. Sgroi , M. Palumbo , P. Rizzi , Pure Appl. Chem. 2024, 96, 511.

[8] M. Palumbo , E. M. Dematteis , L. Fenocchio , G. Cacciamani , M. Baricco , J. Phase Equilib. Diffus. 2024, 45, 273.

[9] E. Callini , Z. Ö. K. Atakli , B. C. Hauback , S.‐I. Orimo , C. Jensen , M. Dornheim , D. Grant , Y. W. Cho , P. Chen , B. Hjörvarsson , P. de Jongh , C. Weidenthaler , M. Baricco , M. Paskevicius , T. R. Jensen , M. E. Bowden , T. S. Autrey , A. Züttel , Appl. Phys. A 2016, 122, 353.

[10] C. Comanescu , Energies 2023, 16, 4536.

[11] F. Habermann , A. Wirth , K. Burkmann , B. Störr , J. Seidel , R. Gumeniuk , K. Bohmhammel , F. Mertens , ChemPhysChem 2024, 25, e202300748. 37963070 10.1002/cphc.202300748

[12] F. Habermann , A. Wirth , K. Burkmann , J. Kraus , B. Störr , H. Stöcker , J. Seidel , J. Kortus , D. C. Meyer , R. Gumeniuk , K. Bohmhammel , F. Mertens , RSC Mechanochem. 2025, 2, 603.

[13] F. Habermann , K. Burkmann , J. Kraus , B. Störr , J. Seidel , K. Bohmhammel , J. Kortus , R. Gumeniuk , F. Mertens , J. Alloys Compd. 2024, 980, 173476.

[14] K. Burkmann , F. Habermann , B. Störr , J. Seidel , R. Gumeniuk , K. Bohmhammel , F. Mertens , RSC Mechanochem. 2025, 2, 563. 10.1002/cphc.202500108 PMC12710114 41223306

[15] K. Burkmann , A. Demmer , F. Habermann , B. Hansel , B. Störr , J. Seidel , R. Gumeniuk , M. Bertau , K. Bohmhammel , F. Mertens , J. Therm. Anal. Calorim. 2025, 150, 5409.

[16] K. Burkmann , F. Habermann , E. Schumann , J. Kraus , B. Störr , H. Schmidt , E. Brendler , J. Seidel , K. Bohmhammel , J. Kortus , F. Mertens , New J. Chem. 2024, 48, 2743.

[17] K. Burkmann , M. Mehlhorn , A. Demmer , J. Kraus , F. Habermann , J. Seidel , K. Bohmhammel , J. Kortus , F. Mertens , Phys. Chem. Chem. Phys. 2025, 27, 17063. 40735935 10.1039/d5cp00744e

[18] J. J. Vajo , F. Mertens , C. C. Ahn , R. C. Bowman , B. Fultz , J. Phys. Chem. B 2004, 108, 13977.

[19] J. J. Vajo , S. L. Skeith , F. Mertens , J. Phys. Chem. B 2005, 109, 3719. 16851415 10.1021/jp040769o

[20] F. Karimi , P. K. Pranzas , C. Pistidda , J. A. Puszkiel , C. Milanese , U. Vainio , M. Paskevicius , T. Emmler , A. Santoru , R. Utke , M. Tolkiehn , C. B. Minella , A.‐L. Chaudhary , S. Boerries , C. E. Buckley , S. Enzo , A. Schreyer , T. Klassen , M. Dornheim , Phys. Chem. Chem. Phys. 2015, 17, 27328. 26418174 10.1039/c5cp03557k

[21] D. J. Siegel , C. Wolverton , V. Ozoliņš , Phys. Rev. B 2007, 76, 134102.

[22] K. T. Møller , A. S. Fogh , M. Paskevicius , J. Skibsted , T. R. Jensen , Phys. Chem. Chem. Phys. 2016, 18, 27545. 27722466 10.1039/c6cp05391b

[23] E. Roedern , T. R. Jensen , J. Phys. Chem. C 2014, 118, 23567.

[24] J. Huang , M. Gao , Z. Li , X. Cheng , J. Gu , Y. Liu , H. Pan , J. Alloys Compd. 2016, 670, 135.

[25] V. Ozolins , E. H. Majzoub , C. Wolverton , J. Am. Chem. Soc. 2009, 131, 230. 19072157 10.1021/ja8066429

[26] S. H. Lee , V. R. Manga , Z.‐K. Liu , Int. J. Hydrogen Energy 2010, 35, 6812.

[27] M. Paskevicius , M. B. Ley , D. A. Sheppard , T. R. Jensen , C. E. Buckley , Phys. Chem. Chem. Phys. 2013, 15, 19774. 24141723 10.1039/c3cp53920b

[28] K. T. Møller , J. B. Grinderslev , T. R. Jensen , J. Alloys Compd. 2017, 720, 497.

[29] E. Dematteis , S. Vaunois , C. Pistidda , M. Dornheim , M. Baricco , Crystals 2018, 8, 90.

[30] E. M. Dematteis , M. Baricco , Energies 2019, 12, 3230.

[31] H. Chu , S. Qiu , L. Sun , G. Wu , J. Renew. Sustainable Energy 2014, 6, 0131051.

[32] S. Soru , A. Taras , C. Pistidda , C. Milanese , C. Bonatto Minella , E. Masolo , P. Nolis , M. D. Baró , A. Marini , M. Tolkiehn , M. Dornheim , S. Enzo , G. Mulas , S. Garroni , J. Alloys Compd. 2014, 615, S693.

[33] S. P. GharibDoust , M. Heere , M. H. Sørby , M. B. Ley , D. B. Ravnsbæk , B. C. Hauback , R. Černý , T. R. Jensen , Dalton Trans. 2016, 45, 19002. 27853777 10.1039/c6dt03671f

[34] E. G. Bardají , Z. Zhao‐Karger , N. Boucharat , A. Nale , M. J. van Setten , W. Lohstroh , E. Röhm , M. Catti , M. Fichtner , J. Phys. Chem. C 2011, 115, 6095.

[35] Y. Chen , R. Sakamoto , A. Inoishi , S. Okada , H. Sakaebe , K. Albrecht , D. H. Gregory , Batteries Supercaps 2024, 7, e202300550.

[36] Z. Yang , N. J. Leon , C. Liao , B. J. Ingram , L. Trahey , ACS Appl. Mater. Interfaces 2023, 15, 25018. 37171170 10.1021/acsami.3c01606

[37] N. T. Hahn , J. Self , T. J. Seguin , D. M. Driscoll , M. A. Rodriguez , M. Balasubramanian , K. A. Persson , K. R. Zavadil , J. Mater. Chem. A 2020, 8, 7235.

[38] L. N. Skov , J. B. Grinderslev , T. S. S. Kjær , L. R. Kristensen , T. R. Jensen , Angew. Chem., Int. Ed. Engl. 2025, 64, e202500613. 39871819 10.1002/anie.202500613 PMC11976212

[39] J. Kollonitsch , O. Fuchs , V. Gabor , Nature 1954, 173, 125.

[40] S. Di Cataldo , L. Boeri , Phys. Rev. B 2023, 107, L060501.

[41] Y. Filinchuk , E. Rönnebro , D. Chandra , Acta Mater. 2009, 57, 732.

[42] M. D. Riktor , M. H. Sørby , K. Chłopek , M. Fichtner , F. Buchter , A. Züttel , B. C. Hauback , J. Mater. Chem. 2007, 17, 4939.

[43] I. Llamas‐Jansa , O. Friedrichs , M. Fichtner , E. G. Bardaji , A. Züttel , B. C. Hauback , J. Phys. Chem. C 2012, 116, 13472.

[44] M. Fichtner , K. Chlopek , M. Longhini , H. Hagemann , J. Phys. Chem. C 2008, 112, 11575.

[45] A. V. Soloninin , O. A. Babanova , A. V. Skripov , H. Hagemann , B. Richter , T. R. Jensen , Y. Filinchuk , J. Phys. Chem. C 2012, 116, 4913.

[46] J.‐H. Kim , J.‐H. Shim , Y. W. Cho , J. Power Sources 2008, 181, 140.

[47] Y. Kim , D. Reed , Y.‐S. Lee , J. Y. Lee , J.‐H. Shim , D. Book , Y. W. Cho , J. Phys. Chem. C 2009, 113, 5865.

[48] Y. Kim , S.‐J. Hwang , J.‐H. Shim , Y.‐S. Lee , H. N. Han , Y. W. Cho , J. Phys. Chem. C 2012, 116, 4330.

[49] Y. Yan , A. Remhof , D. Rentsch , A. Züttel , S. Giri , P. Jena , Chem. Commun. 2015, 51, 11008. 10.1039/c5cc03605d 26008181

[50] Y. Yan , D. Rentsch , A. Remhof , Phys. Chem. Chem. Phys. 2017, 19, 7788. 28262887 10.1039/c7cp00448f

[51] J. Mao , Z. Guo , C. K. Poh , A. Ranjbar , Y. Guo , X. Yu , H. Liu , J. Alloys Compd. 2010, 500, 200.

[52] E. Wiberg , H. Nöth , R. Hartwimmer , Z. Naturforsch. B 1955, 10, 292.

[53] C. Rongeat , V. D’Anna , H. Hagemann , A. Borgschulte , A. Züttel , L. Schultz , O. Gutfleisch , J. Alloys Compd. 2010, 493, 281.

[54] G. Barkhordarian , T. R. Jensen , S. Doppiu , U. Bösenberg , A. Borgschulte , R. Gremaud , Y. Cerenius , M. Dornheim , T. Klassen , R. Bormann , J. Phys. Chem. C 2008, 112, 2743.

[55] E. M. Dematteis , S. R. Jensen , T. R. Jensen , M. Baricco , J. Chem. Thermodyn. 2020, 143, 106055.

[56] V. N. Konoplev , N. N. Mal'tseva , V. S. Khain , Koord. Khim. 1992, 18, 1143.

[57] E. Wiberg , R. Hartwimmer , Z. Naturforsch. B 1955, 10, 294.

[58] E. Wiberg , R. Hartwimmer , Z. Naturforsch. B 1955, 10, 295.

[59] A. Ampoumogli , T. Steriotis , P. Trikalitis , E. G. Bardaji , M. Fichtner , A. Stubos , G. Charalambopoulou , Int. J. Hydrogen Energy 2012, 37, 16631.

[60] B. Richter , J. B. Grinderslev , K. T. Møller , M. Paskevicius , T. R. Jensen , Inorg. Chem. 2018, 57, 10768. 30137973 10.1021/acs.inorgchem.8b01398

[61] E. Rönnebro , E. H. Majzoub , J. Phys. Chem. B 2007, 111, 12045. 17914804 10.1021/jp0764541

[62] V. I. Mikheeva , L. V. Titov , Russ. J. Inorg. Chem. 1964, 9, 440.

[63] V. I. Mikheeva , L. V. Titov , Russ. J. Inorg. Chem. 1964, 9, 437.

[64] L. V. Titov , Dokl. Akad. Nauk SSSR 1964, 154, 654.

[65] A. F. Karabulut , M. Guru , T. A. Boynueğri , M. Y. Aydin , J. Electron. Mater. 2016, 45, 3957.

[66] O. Friedrichs , A. Remhof , A. Borgschulte , F. Buchter , S. I. Orimo , A. Züttel , Phys. Chem. Chem. Phys. 2010, 12, 10919. 20661494 10.1039/c0cp00022a

[67] H. C. Brown , Y. M. Choi , S. Narasimhan , Inorg. Chem. 1982, 21, 3657.

[68] G. Bergerhoff , I. D. Brown , et al., (Ed.: F. H. Allen ), in Crystallographic Databases, International Union of Crystallography, Chester 1987, ICSD.

[69] L. H. Rude , Y. Filinchuk , M. H. Sørby , B. C. Hauback , F. Besenbacher , T. R. Jensen , J. Phys. Chem. C 2011, 115, 7768.

[70] H. Grove , L. H. Rude , T. R. Jensen , M. Corno , P. Ugliengo , M. Baricco , M. H. Sørby , B. C. Hauback , RSC Adv 2014, 4, 4736.

[71] C. G. Maier , K. K. Kelley , J. Am. Chem. Soc. 1932, 54, 3243.

[72] F. Habermann , Synthese Und Thermodynamische Charakterisierung Ausgewählter Alanate, Dissertation, TU Bergakademie Freiberg, Freiberg 2024.

[73] E. R. Pinatel , E. Albanese , B. Civalleri , M. Baricco , J. Alloys Compd. 2015, 645, S64.

[74] A. Roine , HSC Chem. 2002.

[75] E. Gopal , Specific Heats at Low Temperatures, The International Cryogenics Monograph Ser, Springer, New York, NY 1966.

[76] G. Grimvall , Thermophysical Properties of Materials, 1st ed., Elsevier, Amsterdam 1999.

[77] A. Tari , The Specific Heat of Matter at Low Temperatures, Imperial College Press, London and River Edge, NJ 2003.

[78] S. Loos , D. Gruner , M. Abdel‐Hafiez , J. Seidel , R. Hüttl , A. U. Wolter , K. Bohmhammel , F. Mertens , J. Chem. Thermodyn. 2015, 85, 77.

[79] J. M. Schliesser , B. F. Woodfield , Phys. Rev. B 2015, 91, 024109.

[80] K. S. Gavrichev , Inorg. Mater. 2003, 39, S89.

[81] Y.‐S. Lee , J.‐H. Shim , Y. W. Cho , J. Phys. Chem. C 2010, 114, 12833.

[82] K. Miwa , M. Aoki , T. Noritake , N. Ohba , Y. Nakamori , S.‐I. Towata , A. Züttel , S.‐I. Orimo , Phys. Rev. B 2006, 74, 155122.

[83] T. Sato , K. Miwa , Y. Nakamori , K. Ohoyama , H.‐W. Li , T. Noritake , M. Aoki , S.‐I. Towata , S.‐I. Orimo , Phys. Rev. B 2008, 77, 104114.

[84] K. Miwa , N. Ohba , S.‐I. Towata , Y. Nakamori , A. Züttel , S.‐I. Orimo , J. Alloys Compd. 2007, 446–447, 310.

[85] C. Wolverton , V. Ozoliņš , Physical Review B 2007, 75, 064101.

[86] CRC Handbook of Chemistry and Physics: A Ready‐Reference Book of Chemical and Physical Data, 101th ed, (Eds.: J. R. Rumble , T. J. Bruno ), Vol. 2020–2021, CRC Press, Boca Raton, Florida 2020.

[87] Y. Nakamori , K. Miwa , A. Ninomiya , H. Li , N. Ohba , S.‐I. Towata , A. Züttel , S.‐I. Orimo , Phys. Rev. B 2006, 74, 045126.

[88] Y. Nakamori , H. Li , K. Miwa , S.‐I. Towata , S.‐I. Orimo , Mater. Trans. 2006, 47, 1898.

[89] A. J. Du , S. C. Smith , G. Q. Lu , Phys. Rev. B 2006, 74, 193405.

[90] Y. Zhang , E. Majzoub , V. Ozoliņš , C. Wolverton , Phys. Rev. B 2010, 82, 174107.

[91] L.‐L. Wang , D. D. Graham , I. M. Robertson , D. D. Johnson , J. Phys. Chem. C 2009, 113, 20088.

[92] U. Böhme , Inertgastechnik, De Gruyter, Oldenbourg 2020.

[93] K. Burkmann , B. Hansel , F. Habermann , B. Störr , M. Bertau , F. Mertens , Z. Naturforsch. B 2023, 78, 575. 10.1039/d2dt03718a 36942882

[94] D. Thomas , M. Abdel‐Hafiez , T. Gruber , R. Hüttl , J. Seidel , A. U. Wolter , B. Büchner , J. Kortus , F. Mertens , J. Chem. Thermodyn. 2013, 64, 205.

[95] D. Thomas , N. Bette , F. Taubert , R. Hüttl , J. Seidel , F. Mertens , J. Alloys Compd. 2017, 704, 398.

[96] F. Taubert , S. Schwalbe , J. Seidel , R. Hüttl , T. Gruber , R. Janot , M. Bobnar , R. Gumeniuk , F. Mertens , J. Kortus , Int. J. Mater. Res. 2017, 108, 942.

[97] F. Taubert , J. Seidel , R. Hüttl , M. Bobnar , R. Gumeniuk , F. Mertens , J. Chem. Thermodyn. 2018, 116, 323.

[98] F. Taubert , J. Seidel , R. Hüttl , M. Bobnar , R. Gumeniuk , F. Mertens , J. Chem. Thermodyn. 2019, 130, 119.

[99] F. Taubert , D. Thomas , R. Hüttl , J. Seidel , F. Mertens , J. Alloys Compd. 2022, 897, 163147.

[100] H. M. Rietveld , Acta Cryst. 1967, 22, 151.

[101] H. M. Rietveld , J. Appl. Crystallogr. 1969, 2, 65.

[102] A. A. Coelho , Topas 2014, 5.

[103] K. Burkmann , F. Habermann , R. Gumeniuk , F. Mertens , Z. Naturforsch. B 2024, 79, 293.

[104] C. A. Kennedy , M. Stancescu , R. A. Marriott , M. A. White , Cryogenics 2007, 47, 107.

[105] J. C. Lashley , M. F. Hundley , A. Migliori , J. L. Sarrao , P. G. Pagliuso , T. W. Darling , M. Jaime , J. C. Cooley , W. L. Hults , L. Morales , D. J. Thoma , J. L. Smith , J. Boerio‐Goates , B. F. Woodfield , G. R. Stewart , R. A. Fisher , N. E. Phillips , Cryogenics 2003, 43, 369.

[106] S. Loos , Olivin‐Typ Lithiumeisenphosphat (Li1‐XFePO4) ‐ Synthese, Li‐Ionentransport Und Thermodynamik, Dissertation, TU Bergakademie Freiberg, Freiberg 2014.

[107] Q. Shi , C. L. Snow , J. Boerio‐Goates , B. F. Woodfield , J. Chem. Thermodyn. 2010, 42, 1107.

[108] W. Schnelle , J. Engelhardt , E. Gmelin , Cryogenics 1999, 39, 271.

[109] C. A. Swenson , Rev. Sci. Instrum. 1999, 70, 2728.

[110] G. K. White , S. J. Collocott , J. Phys. Chem. Ref. Data 1984, 13, 1251.

[111] S. M. Sarge , G. W. H. Höhne , W. Hemminger , Calorimetry: Fundamentals, Instrumentation and Applications, Wiley‐VCH Verlag, Weinheim, Germany 2014.

[112] D. Thomas , M. Zeilinger , D. Gruner , R. Hüttl , J. Seidel , A. U. Wolter , T. F. Fässler , F. Mertens , J. Chem. Thermodyn. 2015, 85, 178.

[113] G. Della Gatta , M. J. Richardson , S. M. Sarge , S. Stølen , Pure Appl. Chem. 2006, 78, 1455.

[114] C. W. Bale , E. Bélisle , P. Chartrand , S. A. Decterov , G. Eriksson , A. E. Gheribi , K. Hack , I. H. Jung , Y. B. Kang , J. Melançon , A. D. Pelton , S. Petersen , C. Robelin , J. Sangster , P. Spencer , M. A. van Ende , Calphad 2016, 54, 35.

